# Food‐web dynamics of a floodplain mosaic overshadow the effects of engineered logjams for Pacific salmon and steelhead

**DOI:** 10.1002/eap.3076

**Published:** 2024-12-03

**Authors:** James C. Paris, Colden V. Baxter, J. Ryan Bellmore, Joseph R. Benjamin

**Affiliations:** ^1^ Department of Biological Sciences Idaho State University Pocatello Idaho USA; ^2^ Pacific Northwest Research Station, US Forest Service Juneau Alaska USA; ^3^ Forest and Rangeland Ecosystem Science Center, US Geological Survey Boise Idaho USA

**Keywords:** engineered logjams, food webs, Pacific salmon, river restoration, river‐floodplains, secondary production, shifting habitat mosaic, trophic basis of production

## Abstract

Food webs vary in space and time. The structure and spatial arrangement of food webs are theorized to mediate temporal dynamics of energy flow, but empirical corroboration in intermediate‐scale landscapes is scarce. River‐floodplain landscapes encompass a mosaic of aquatic habitat patches and food webs, supporting a variety of aquatic consumers of conservation concern. How the structure and productivity of these patch‐scale food webs change through time, and how floodplain restoration influences their dynamics, are unevaluated. We measured productivity and food‐web dynamics across a mosaic of main‐channel and side‐channel habitats of the Methow River, WA, USA, during two study years (2009–2010; 2015–2016) and examined how food webs that sustained juvenile anadromous salmonids responded to habitat manipulation. By quantifying temporal variation in secondary production and organic matter flow across nontreated river‐floodplain habitats and comparing that variation to a side channel treated with engineered logjams, we jointly confronted spatial food‐web theory and assessed whether food‐web dynamics in the treated side channel exceeded natural variation exhibited in nontreated habitats. We observed that organic matter flow through the more complex, main‐channel food web was similar between study years, whereas organic matter flow through the simpler, side‐channel food webs changed up to ~4‐fold. In the side channel treated with engineered logjams, production of benthic invertebrates and juvenile salmonids increased between study years by 2× and 4×, respectively; however, these changes did not surpass the temporal variation observed in untreated habitats. For instance, juvenile salmonid production rose 17‐fold in one untreated side‐channel habitat, and natural aggregation of large wood in another coincided with a shift to community and food‐web dominance by juvenile salmonids. Our findings suggest that interannual dynamism in material flux across floodplain habitat mosaics is interrelated with patchiness in food‐web complexity and may overshadow the ecological responses to localized river restoration. Although this dynamism may inhibit detection of the ecological effects of river restoration, it may also act to stabilize aquatic ecosystems and buffer salmon and other species of conservation concern in the long term. As such, natural, landscape‐level patchiness and dynamism in food webs should be integrated into conceptual foundations of process‐based, river restoration.

## INTRODUCTION

Food webs, which map the pathways of material and energy flowing through organisms and ecosystems, fluctuate naturally in space and time. This spatiotemporal variability is key for the persistence of complex communities and species of interest that are embedded in Earth's landscapes (de Ruiter et al., 2005; McCann et al., 2006; Polis et al., 2004; Polis & Strong, 1996). Although theory and models have made strides toward combining landscape‐level thinking with food webs by illustrating how spatial variation in food‐web structure can mediate food‐web behavior and stability (Rooney et al., 2008; Rooney & McCann, 2012), few studies have empirically explored these ideas in landscapes of intermediate scale (i.e., 10^3^–10^5^ m). Furthermore, perspectives involving spatial variation and temporal dynamics of food webs rarely inform or evaluate ecological restoration, which is on the rise in river landscapes (Bernhardt et al., 2005; Palmer & Ruhi, 2019; Wohl et al., 2015) or “riverscapes” (sensu Fausch et al., 2002; Torgersen et al., 2022). River restoration often aims to restore static habitat features or the processes that create and maintain physical habitat condition (Beechie et al., 2010; Wohl et al., 2005), with the assumption that food resources and/or bioenergetic carrying capacity to support organisms will subsequently increase (Wipfli & Baxter, 2010), but how the structure and dynamics of food webs actually vary and respond to river restoration are generally neglected (Naman et al., 2022). Studies that quantify and illuminate riverscape food‐web heterogeneity and dynamics within the context of river restoration, therefore, can jointly confront emerging theory with empirical findings as well as inform ecological restoration by examining links between aquatic food‐web dynamics and restoration practice.

River‐floodplain ecosystems within riverscapes are spatially complex, highly dynamic, yet imperiled by human degradation. Within gravel‐bedded floodplains, streamflow, sediment, and riparian vegetation interact to create cycles of erosion and deposition, which together give rise to a constantly shifting mosaic of channels, bars, islands, benches, and ponds (Hauer et al., 2016; Stanford et al., 2005; Wohl, 2013). As a result of such patchiness and dynamism in habitat, the abundance and distribution of organisms across river‐floodplain mosaics vary in space and time (Harner & Stanford, 2003; Mouw et al., 2013; Robinson et al., 2002). Past research has shown that community composition and food‐web structure differ across aquatic habitats of alluvial floodplain mosaics (Bellmore et al., 2013), which makes gravel‐bed river‐floodplains fertile locations to explore theory related to food‐web heterogeneity and dynamism (Tockner, Lorang, et al., 2010; Tockner, Pusch, et al., 2010). Although river‐floodplains provide many ecosystem services and sustain species of interest, floodplains worldwide have been leveed, dammed, farmed, and developed (Tockner, Lorang, et al., 2010; Tockner, Pusch, et al., 2010), resulting in widespread disconnection of rivers from their floodplains, homogenization of floodplain mosaics, and, subsequently, the use of habitat manipulation intended to reverse degradation (Bayley, 1991; Bernhardt et al., 2005; Pess et al., 2005). River‐floodplain mosaics are thus effective settings for merging the study of food‐web dynamics across patchy habitat mosaics with assessments of habitat manipulation.

Pacific salmon (genus *Oncorhynchus*) are embedded within food‐web dynamics in freshwaters and are common targets of habitat manipulation in rivers. A large body of research has illuminated the many ways Pacific salmon populations influence and are influenced by freshwater food webs (Gende et al., 2002; Naiman et al., 2002; Schindler et al., 2003; Wipfli & Baxter, 2010). It is relatively unknown, however, the degree to which they contribute and respond to productivity and food‐web dynamics across riverine mosaics, although they rely on habitat mosaics at various scales to complete their life cycle (Baldock et al., 2016; Brennan et al., 2019). Thus, more spatially explicit food‐web studies encompassing salmon in habitat mosaics may be beneficial. Such studies may be useful in floodplains of the Columbia River Basin, USA, where habitat manipulation is commonly employed to engineer productive rearing habitat and mitigate for continued mortality at other life stages (Beechie et al., 2013; Bond et al., 2019; Budy & Schaller, 2007). In general, these habitat enhancement efforts occur at small scales and often utilize engineered logjams (see Pess et al., 2012; Roni et al., 2015). Yet, food‐web methods are rarely used to evaluate such efforts (Bellmore et al., 2017; Naiman et al., 2012), and, more specifically, few studies that evaluate habitat manipulation effects on salmon consider the broader context of food‐web dynamics in patchy riverscapes—across which juvenile salmon are widely distributed (Rossi et al., 2024).

Here, we investigated food‐web dynamics across a river‐floodplain mosaic and compared these dynamics to what was observed in a restored side‐channel habitat treated with engineered logjams. To do so, we replicated food‐web sampling and analyses conducted by Bellmore et al. (2013, 2015) in the Methow River, WA, which surveyed invertebrate and fish communities, estimated secondary production, and built organic matter flow food webs in the main river channel and three side‐channel habitats. We then made comparisons between two study years, which occurred 5 years apart (2009–2010 and 2015–2016); between study years, one side‐channel habitat was treated with engineered logjams, whereas the main channel and remaining side‐channel habitats were not treated. By juxtaposing food‐web dynamics observed in the nontreated, reference habitats to those of the treated side‐channel habitat, we assessed whether habitat manipulation produced detectable effects on food‐web dynamics and salmon production against the backdrop of natural variability.

We had two research questions. First, how do secondary production of benthic invertebrates and fishes and the pathways of organic matter flow supporting fishes change through time across the main channel and multiple side‐channel habitats? Based on spatial food‐web theory related to distinct “energy channels” across landscapes (Rooney et al., 2008; Rooney & McCann, 2012), we expected that the more species‐rich and reticulate food web of the main channel would be more stable (i.e., resistant to change) through time than the simpler food webs of the side channels. Second, does the magnitude of change in food‐web structure and productivity within the side channel treated with engineered logjams surpass natural variability observed in untreated sites? Specifically, relative to untreated habitats, was the engineered logjam treatment associated with (1) increased organic matter flow to fishes, especially juvenile Chinook salmon (*Oncorhynchus tshawytscha*), coho salmon (*Oncorhynchus kisutch*), and steelhead trout (*Oncorhynchus mykiss*); (2) more efficient transfer of benthic invertebrate production to fish consumption; and (3) shifts in food‐web interaction strengths? Based on analyses by Bellmore et al. (2013), which suggested side‐channel habitats of the Methow River floodplain were “under‐seeded” with respect to food (i.e., below estimated carrying capacity), we expected that habitat engineering might increase invertebrate production but would not increase the magnitude or efficiency of material flow to fishes in the treated side‐channel. Our findings reveal a shifting mosaic of food webs across the river floodplain and suggest that natural interannual variation in food‐web structure may exceed the ecological responses to localized river restoration.

## METHODS

### Study site and habitats

**FIGURE 1:**
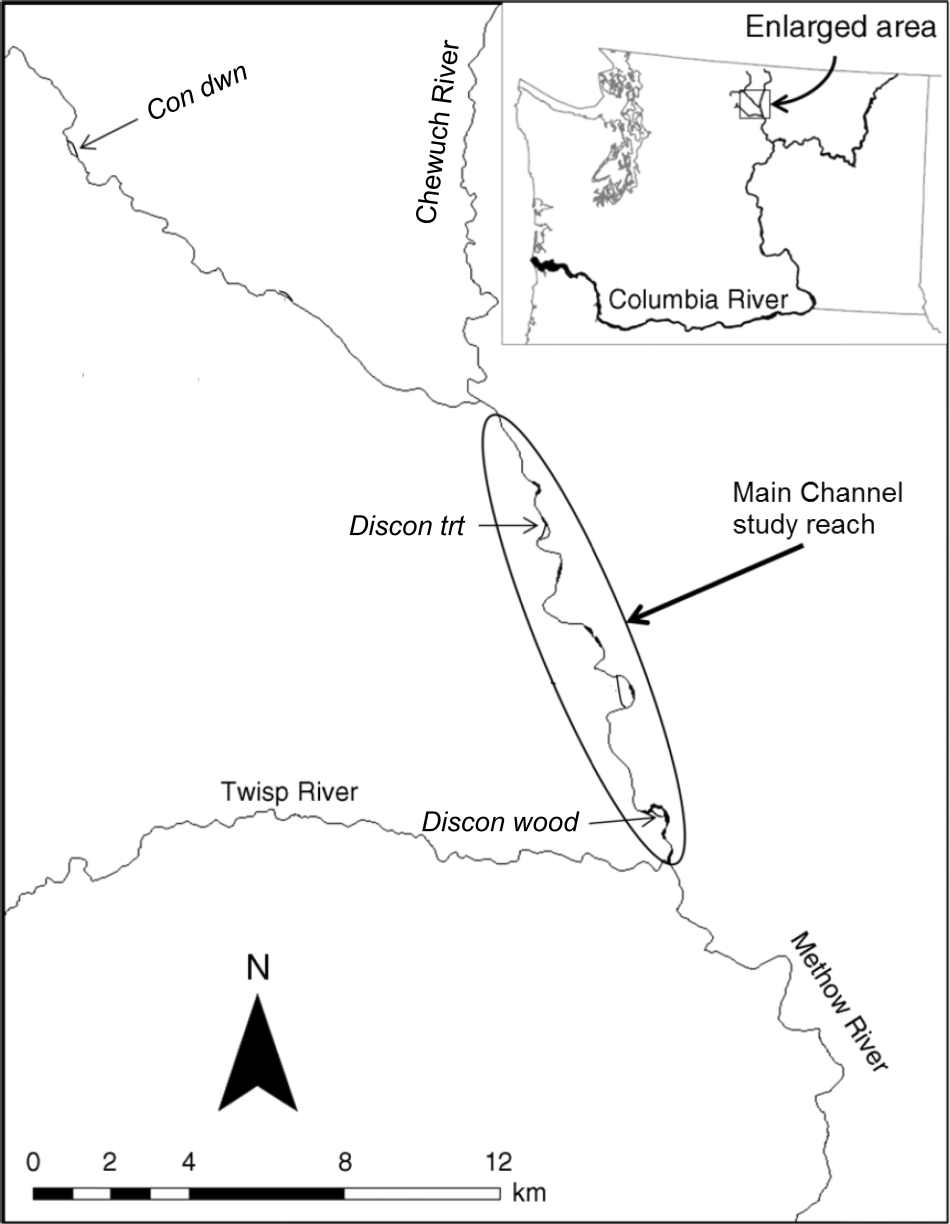
Map of the Methow River, WA, USA, and study sites sampled during study year 1 (2009–2010) and study year 5 (2015–2016). Inset shows the location of Methow River subbasin within Columbia River basin. See *Methods* for hydrogeomorphic description of side‐channel habitats. *con dwn*, connected downstream; *discon trt*, disconnected treated with engineered logjams; *discon wood*, disconnected with natural large wood.

**FIGURE 2:**
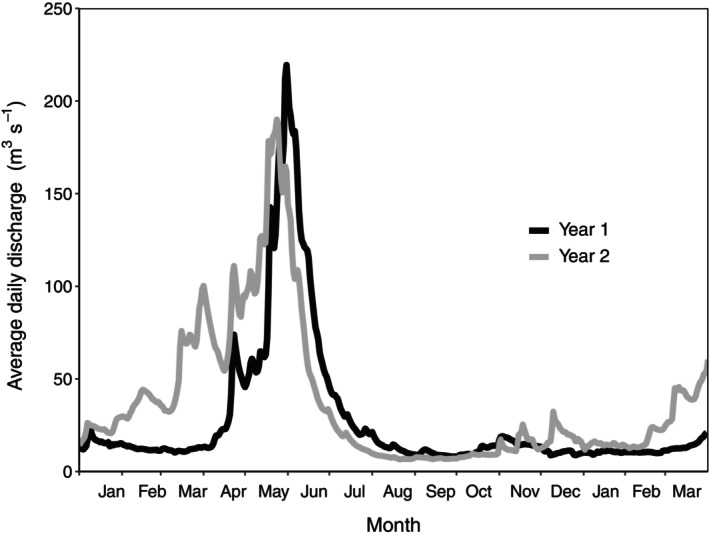
Average daily discharge of the Methow River, WA, USA, near its confluence with the Columbia River (USGS gage 12449950) during study year 1 (2009–2010, black line) and study year 5 (2015–2016, gray line).

The Methow River, a fifth‐order tributary of the Columbia River in North‐Central Washington, USA, drains the eastern Cascade Range and flows southeast for nearly 100 km from 1700 to 240 m elevation; the Chewuch River and the Twisp River are principal tributaries (Figure 1). The watershed encompasses 4662 km^2^ of semi‐arid, mountainous terrain, and winter snow forms the majority of catchment water yield, with average precipitation amounting to 81 cm year^−1^ (Walters & Nassar, 1974). Peak discharge follows spring‐runoff (April–June), reaching magnitudes of 300 m^3^ s^−1^ at the river's mouth (U.S. Geological Survey, 2020), and groundwater discharge supplies baseflow in late summer and fall (August–November; see Figure 2 as an example). In multiple locations, the Methow River occupies glacially scoured valleys filled with coarse‐grained alluvium and forms broad, gravel‐bed floodplains (Ely, 2003). A 15‐km segment of river‐floodplain located between confluences of the Chewuch and Twisp Rivers was targeted for manipulation to enhance side‐channel rearing habitat for juvenile Chinook and coho salmon and steelhead trout (Bellmore et al., 2013).

The Methow River is free of large impoundments, but nine major dams exist on the mainstem Columbia River between its confluence with the Methow River and the Pacific Ocean. Once plentiful runs of Chinook salmon, coho salmon, and steelhead trout to the basin have declined since European settlement (French & Wahle, 1960). At present, spring Chinook salmon and summer steelhead in the Columbia River basin are listed as endangered or threatened, respectively, under the U.S. Endangered Species Act of 1973. Coho salmon were extirpated from the drainage in the 1920s, but now spawn in low abundance following hatchery reintroduction (Galbreath et al., 2014). During the time of these studies, Chinook salmon and coho salmon built 2.4× and 4.5× more redds (i.e., nests where salmon bury their eggs in gravel substrate), respectively, in the main channel in 2014 than in 2008 (Snow et al., 2017; G. Watson, personal communication). Thus, we infer that adult returns to the Methow River basin were greater in the year prior to the 2015–2016 sampling period than in the year prior to the 2009–2010 sampling period. Westslope cutthroat trout (*Oncorhynchus clarkii lewisi*), mountain whitefish (*Prosopium williamsoni*), bull trout (*Salvelinus confluentus*; endangered under Endangered Species Act), rainbow trout (*O. mykiss*), bridgelip sucker (*Catostomus columbianus*), longnose dace (*Rhinichthys cataractae*), and at least three species of sculpin (*Cottus bairdii*, *Cottus confusus*, *Cottus rhotheus*) form the rest of the fish community. Non‐native brown bullhead (*Ameiurus nebulosus*), smallmouth bass (*Micropterus dolomieu*), and brook trout (*Salvelinus fontinalis*) have been observed in the basin but were rare at the time and place of this study. Benthic invertebrate communities are comprised of principally native aquatic insect taxa.

We studied four river‐floodplain habitats that encompassed a range of hydrogeomorphic conditions (Appendix S1). Bellmore et al. (2013) initially studied these habitats in a comparative food‐web study, and field sampling occurred at all sites from summer 2009 to summer 2010 (hereafter year 1) and was repeated from summer 2015 to summer 2016 (hereafter year 5; Figure 1). This included three reference habitats (i.e., no restoration treatment): (1) an 8‐km reach of the main river channel (hereafter *main ch*), (2) a 0.8‐km side channel that maintained year‐round connectivity with the main channel at its downstream end during both study years (hereafter *con dwn*), and (3) a 0.6‐km side channel that was an open, isolated pool during year 1 baseflow, but a natural logjam developed between study years and shifted wetted habitat to beneath the logjam (hereafter *discon wood*).

The 0.6‐km, treated side channel (hereafter *discon trt*) experienced upstream and downstream disconnection during year 1 (Bellmore et al., 2013). In 2013, seven engineered logjams were installed along its length, and gravel was excavated and redistributed to promote connectivity with the main channel and maintenance of riffle‐pool habitat throughout the year.

All side channel sites were located within the parafluvial zone of the floodplain, and floods during spring runoff connected them to the main channel and delivered sediment and large wood. Onset HOBO (Onset Corp., Bourne, MA) temperature loggers were deployed to record temperature at all sites during both study periods, except during high flows.

### Invertebrate sampling and estimates of production

We quantitatively sampled aquatic benthic invertebrates at each site during year 1 and year 5 to estimate annual biomass and secondary production. We seasonally collected samples using a modified, shovel‐handled Surber net (250‐μm mesh; 0.26 m^2^ sample area; see Bellmore et al., 2013). During year 1, we collected subsamples (*n* = 3–11) from different channel units (e.g., pools, riffles, glides) in proportion to their occurrence within the total habitat. We combined subsamples from each site to generate multiple replicate composite samples at each site (*n* = 3–5) in order to encompass spatial variation in invertebrate abundance across the total habitat, while reducing the number of samples analyzed. During year 5, we collected subsamples (*n* = 3–5) from multiple equally spaced channel cross sections (*n* = 3–5) along the length of a given habitat by randomly generating a number and then sampling along a given cross section at a distance, in meters, from the bank equal to the random number (i.e., 15 would equal 15 m from the bank). The subsamples from each cross section were combined in order to create a replicate composite sample at each site (*n* = 3–5 composite samples). Although the ways in which subsamples were stratified and sampled within habitats differed between the two sampling years, and therefore, the same sub‐habitats (i.e., channel units) were not perfectly represented in year 1 and year 5, we judged that both methodologies used would capture some degree of the spatial heterogeneity present in the composition and biomass of invertebrate communities across different sub‐habitats. For this study, we assumed such spatial heterogeneity has the potential to contribute greater variation to our measurements than the variation attributable to different methods used. Thus, we judged that reflecting spatial heterogeneity in invertebrate presence and abundance in sampling was more important than maintaining exact methodological consistency. In flowing habitats, one person held the Surber net in place, while another disturbed the benthic surface to a depth of 10 cm. When side channels became disconnected during baseflow, the person disturbing sediment also hand‐scooped water through the net to deliver invertebrates. Organic matter and invertebrates from composite samples were elutriated to remove sediment, poured through a 250‐μm sieve, and preserved in 95% ethanol.

We removed invertebrates from each composite sample in the laboratory using a two‐phased approach (Vinson & Hawkins, 1996). Large individuals (>10 mm) were first picked from the organic matter and sorted. Then, we successively removed subsamples from the composite sample and picked invertebrates under 10× magnification until we removed 500 individuals. Invertebrates were identified to species when possible and counted. We estimated per‐area abundance (in individuals per square meter) by multiplying counts by the inverse of the subsample percentage and then dividing by Surber quadrat area. To estimate dry biomass of invertebrate taxa (in grams of dry matter per square meter) during year 1, invertebrates were dried at 60°C for 24 h and weighed. During year 5, the total lengths of individual invertebrates were measured to the nearest millimeter, and literature‐derived length‐mass regressions were applied to estimate body mass (Benke et al., 1999). Average annual biomass of the invertebrate assemblage at each site was calculated by summing biomass of taxa and averaging across seasonal samples. We estimated 95% CIs by bootstrapping the taxon‐specific biomass estimates, whereby we resampled biomass estimates from all seasonally collected replicates from a given site with replacement 1000 times and created 97.5% and 2.5% quantiles for average annual biomass (Bellmore et al., 2013; Benke & Huryn, 2017; Cross et al., 2013).

We estimated annual secondary production (in grams of dry matter per square meter per year) of benthic invertebrates using multiple methods, depending on taxon identity and life history characteristics. For taxa with distinct population size structure, we used the size‐frequency method corrected for our best estimates of cohort production interval (CPI; Benke & Huryn, 2017). During year 1, we calculated production at the main channel and one side channel using additional composite samples collected each month to generate size structure data and CPIs for each taxon. Production estimates for each taxon in the monthly sampled main‐channel and side‐channel habitats were divided by average annual biomass to arrive at production to biomass ratios (P:B, per year), or annual rates of biomass turnover, which were then multiplied by annual biomass of corresponding taxa in the other sites (see Bellmore et al., 2013 for further description and rationale). During year 5, monthly samples were not collected, and we relied on invertebrate size distributions generated from seasonal biomass samples to calculate CPIs.

To calculate production by Chironomidae larvae, which can have multiple generations a year with overlapping cohorts, we separated individuals into three dominant subfamilies: Chironominae, Orthocladiinae, and Tanypodinae. During both study years, we averaged estimates derived from the size‐frequency method using literature‐based CPI and from the instantaneous growth method using size‐ and temperature‐specific equations from Huryn (1990) and Walther et al. (2006). For rare taxa, we multiplied annual P:B ratios from streams in the Pacific Northwest (Gaines et al., 1992; Robinson & Minshall, 1998) to annual biomass estimates. Total annual production for each site was the sum of taxon‐specific production estimates. To estimate 95% CIs for annual production, we utilized a bootstrapping approach (Benke & Huryn, 2017), whereby we multiplied taxon‐specific P:B ratios by the estimates of the 97.5% and 2.5% quantiles of average annual biomass developed for each taxon such that each taxon possessed upper and lower quantiles for 1000 estimates of annual biomass values generated through randomly resampling the biomass values.

### Fish surveys and estimates of production

Fish assemblages at each site were surveyed to estimate abundance, growth, and species composition. Combinations of snorkeling and electrofishing conducted by the Bureau of Reclamation and local partners were utilized to maximize detection probability within different habitat types (Baxter et al., 2019). In side‐channel habitats, fish surveys were conducted in June, August, October, and March of year 1 and in July, August, October, and March of year 5. Multi‐pass electrofishing surveys in block‐netted channel units were used to estimate fish abundance via the removal‐depletion method (Connolly, 1996). For instances with inadequate depletion, we applied species‐ and channel‐unit‐specific capture efficiencies from other time periods. When depletion surveys were logistically difficult (deep pools, high flows, complex habitat), we used a combination of electrofishing and snorkeling. However, this often increased the error surrounding estimates of fish abundance, especially where capture and observation efficiencies were low (e.g., *discon wood*). We recorded length and mass of captured fishes, and juvenile salmonids were implanted with a passive integrated transponder (PIT) tag to estimate growth rates of recaptured individuals.

In the main channel, snorkel surveys were conducted approximately monthly during year 1 and seasonally during year 5. Multiple observers drifted downstream over the 8‐km study reach and counted adult mid‐water‐column fishes and separated fish into size classes (size classes: 150–300 mm, 300–500 mm, and >500 mm). Error in snorkel surveys was estimated by repeating downstream surveys over three consecutive days. We converted snorkel counts to abundance estimates by applying an observation efficiency derived from a mark‐resight effort involving mountain whitefish (year 1: *n* = 30 whitefish, observation efficiency of 0.4; year 5: *n* = 20 whitefish, observation efficiency of 0.3). Single‐pass electrofishing was conducted seasonally in 400–800 m sections of river margin to capture juvenile salmonids (<150 mm) and longnose dace. We applied capture efficiencies associated with electrofishing surveys in the side channels to calculate abundance. Abundance of sculpin was estimated in three randomly selected riffles and runs in summer 2010 by electrofishing within a Surber quadrat. During year 5, however, sculpin were only enumerated during the single‐pass electrofishing in the river margins, and juvenile sculpin were rarely detected (~10% of captured individuals were <40 mm in length). Given that juveniles composed a substantial fraction of sculpin populations in the main channel during year 1 (~60% were <40 mm) and juvenile sculpin are extremely difficult to capture without targeted search, we assumed that fewer juvenile sculpin was the result of inefficiency in their capture and applied densities of juvenile sculpin from year 1. While this likely produced error in our estimates, we assumed that a larger source of uncertainty stemmed from lumping multiple sculpin species for analysis. Sculpin were not identified to species in the original study, and thus, doing so during year 5 was impractical.

To estimate per‐area biomass (in grams of dry matter per square meter) of each fish species on each sampling date, we multiplied size‐ and species‐specific abundance estimates by average mass and divided by sampling area (Hayes et al., 2007). Average mass of individuals, regardless of methodologies used to estimate abundance, ultimately originated from captured and handled fish. In the side channels and main channel margins, such weighing occurred during electrofishing surveys, and for mid‐water column fish in the main channel, weighing occurred after fish were captured via angling to collect stomach content samples. For each species, we developed length‐mass regressions curves from all the weighed, captured individuals, which were then used to derive average masses for multiple binned size classes (size classes: 150–300 mm, 300–500 mm, and >500 mm). We converted wet biomass to dry biomass by assuming 80% water content for juvenile fishes and 75% for adult fishes and sculpin (Bellmore et al., 2013). Summing dry biomass of each size class within a habitat yielded total seasonal biomass for a given species, and averaging seasonal biomass values yielded mean annual biomass. We estimated secondary production (in grams of dry matter per square meter per year) of fishes through a combination of methods. For juvenile salmonids, we used the instantaneous growth rate method (Hayes et al., 2007), whereby average annual growth rates for each species and size class were derived from recaptured PIT‐tagged individuals, which we multiplied by annual biomass estimates. For larger size classes (>150 mm), we used the cohort production method (Hayes et al., 2007). Due to low abundances of juvenile coho during spring and summer of year 5, we relied on a combination of growth rate and cohort methods for this species. Production of sculpin was estimated using the size‐frequency method (Hayes et al., 2007) based on the size‐structure and abundance at each site and was corrected by an assumed CPI of 5 years. Production of longnose dace was estimated using an annual growth rate from Neves and Pardue (1983). In the main channel, we multiplied annual growth rates from Bellmore et al. (2013) by biomass estimates of adult fishes from year 5. This may have led to error in our production estimates, but we assume the largest source of uncertainty originated in the downstream snorkel surveys and mark‐resight methods. If growth rates for adult fishes varied between time periods, we expected this range of variation would be smaller than the possible range of variation in biomass estimates. Similarly, we used annual growth rates from Bellmore et al. (2013) for bridgelip suckers, which were rare during year 5. We calculated error around secondary production by propagating standard errors of annual biomass and fish growth rate (Taylor, 1997).

### Food‐web analyses

We used the flow food‐web approach (Benke & Huryn, 2017; Benke & Wallace, 1997) to quantify organic matter flows from invertebrates to fishes (i.e., rates of fish consumption). This method requires (1) estimates of secondary production of fishes, (2) diet fractions of fishes attributed to different prey taxa, and (3) bioenergetic efficiencies. Using these metrics, we calculated the relative and absolute contribution of each prey taxon to fish production (i.e., trophic basis of production) and the estimated rates of consumption needed to support observed levels of fish production (i.e., organic matter flow). We calculated the efficiency of organic matter transfer to fishes (i.e., ecotrophic efficiency) by dividing estimates of fish consumption of aquatic invertebrates by total invertebrate secondary production.

To quantify fish diet composition at each site, we seasonally sampled gut contents from dominant members of the fish assemblage (year 1: *n* = 375; year 5: *n* = 645); more samples were taken in year 5 due to higher fish abundances in multiple habitats. Fish were collected for diet sampling in the side channels and main‐channel margins during electrofishing surveys, and adult fishes in the main channel were collected via angling. Nonlethal gastric lavage was used to flush stomach contents of all salmonids >75 mm into a 250‐μm sieve. Stomach contents were then preserved in 95% ethanol. Sculpin were lavaged during year 1, but sculpin stomach contents were dissected from euthanized individuals during year 5. During both study years, longnose dace and bridgelip suckers were sacrificed and their foreguts (first 10% of digestive tract) were removed. Invertebrates in diet samples were identified to genus and species when possible, and total body lengths were converted to biomass (in grams of dry matter) using published length‐mass regressions (Benke et al., 1999). We quantified diet composition by calculating the proportion that each prey taxon contributed to total biomass of gut contents. We primarily quantified diet proportions composed of invertebrate prey, as a main motivation for this set of studies was to compare rates of consumption by fishes to rates of production by invertebrate prey across space and through time; thus, we generally omitted materials and items from analysis that lacked production estimates (i.e., aquatic and terrestrial detritus, biofilms and periphyton, salmon eggs). However, such prey items rarely formed substantial proportions of fish diets during the time of our study. Mean annual diet composition of each fish species at each site was calculated by averaging diet proportions of all individuals across seasons. For food‐web analyses, we lumped invertebrate taxa at the family level to reduce error derived from difficulty in identifying digested individuals to genus or species.

We estimated trophic basis of fish production (Benke and Wallace, 1980) as follows: The relative fraction (*F*_*i*_) that a prey resource *i* contributed to annual production of fish species *j* was calculated as follows:

(1) $$F_{i}=G_{i}\times {\mathrm{AE}}_{\mathrm{ij}}\times {\mathrm{NPE}}_{j}$$

where *G*_*i*_ is the average annual diet proportion attributed to prey item *i*, AE_*ij*_ is the assimilation efficiency of prey item *i* by fish consumer *j* (i.e., the proportion of digestible material), and NPE_*j*_ is the net production efficiency of fish consumer *j* (i.e., proportion of assimilated material used in production). The proportion of production by fish consumer *j* derived from prey item *i* (PF_*ij*_) was calculated as follows:

(2) $${\mathrm{PF}}_{\mathrm{ij}}=\frac{F_{i}}{\sum_{i=1}^{n}F_{i}}$$

Rates of organic matter flow (in grams of dry matter per square meter per year) of prey item *i* to fish *j* (FC_*ij*_) were estimated as follows:

(3) $${\mathrm{FC}}_{\mathrm{ij}}=\frac{{\mathrm{PF}}_{\mathrm{ij}}\times P_{j}}{{\mathrm{AE}}_{i}\times \mathrm{NPE}}$$

where *P*_*j*_ is the annual secondary production of fish *j* (in grams of dry matter per square meter per year). Replicating Bellmore et al. (2013), we used the following assimilation efficiencies for diet items consumed by salmonids: 0.75 for aquatic invertebrates, 0.70 for terrestrial arthropods, and 0.90 for fish tissue. Assimilation efficiencies of diet items for non‐salmonids, which predominately consumed aquatic invertebrates, were 0.90 for longnose dace, 0.85 for bridgelip sucker, and 0.82 for sculpin species. To account for ontogenetic shifts in consumption–growth relationships, we used a net production efficiency of 0.25 for juvenile salmonids and non‐salmonid fishes and 0.125 for adult salmonids (Bellmore et al., 2013; Cross et al., 2011; Donner, 2011). We calculated response ratios (magnitude of year 5 flow divided by magnitude of year 1 flow) for dominant organic matter flow pathways in order to construct “change webs.”

A variety of methods can estimate the strength of food‐web interactions (IS; Wootton & Emmerson, 2005), and we followed past studies from the Methow River (Bellmore et al., 2013, 2015) by using the following equation:

(4) $$\text{IS}=\frac{{\mathrm{FC}}_{\mathrm{ij}}}{P_{i}}$$

We estimated ecotrophic efficiency (i.e., the proportion of total benthic invertebrate production consumed by the fish assemblage over the course of the year) at each site by dividing total annual organic matter flow to fishes by the total annual production of aquatic invertebrates in each site (Christensen & Walters, 2004).

### Analytical design

To evaluate responses to the engineered logjam treatment, we compared food‐web dynamics between year 1 and year 5 among all sites. We employed a modified Before‐After‐Control‐Impact (BACI) framework (Stewart‐Oaten et al., 1992), whereby changes in secondary production, organic matter flow, and food‐web structure across the three non‐manipulated study sites (*main ch*, *con dwn*, and *discon wood*) represented the range of natural food‐web variability present in the Methow River floodplain. This representation was a partial snapshot of the full range of possible variation; additional sites and time periods would need to have been surveyed in order to better approximate total natural variability. Food‐web changes at the treated side channel (*discon trt*) were compared with those in the non‐manipulated sites such that if the magnitude of change in a given estimate (e.g., production, organic matter flow, interaction strengths) at *discon trt* exceeded what was observed at one or more of the unmanipulated sites, we concluded the engineered logjams drove an ecologically relevant response. In some cases, the specific methods of sample collection, processing, and analyses differed between study years. We assumed, however, that seasonal variation in invertebrates, fishes, and their feeding interactions—which we aimed to integrate into analysis through annualized estimates of biomass, production, and organic matter flow—outweighed error that might arise from such methodological discrepancies.

For estimates with associated uncertainty (i.e., secondary production, total organic matter flow), we visually compared 95% CIs among sites, with ecologically relevant differences defined by non‐overlapping CIs. For estimates with very large 95% CIs that overlapped with zero (e.g., taxon‐specific rates of secondary production), we assumed relevant changes as those of least an order of magnitude. Given our analytical approach, we avoided “statistically significant” and “statistically non‐significant” designations, consistent with current statistical guidance, norms, and consensus (Wasserstein et al., 2019). For food‐web attributes lacking estimates of uncertainty (e.g., food‐web complexity, connectance, ecotrophic efficiency, and interaction strengths), we visually compared the direction and magnitude of changes between study years and relied on multiple lines of evidence weighted by magnitudes of change.

## RESULTS

### Water temperature and discharge

Water temperature seasonally varied across study sites (Appendix S2), and the magnitude of winter and spring flows of the Methow River differed between study years (Figure 2). Average annual temperatures were similar between years at all sites. Fall and winter temperatures were slightly warmer at *main ch* and *con dwn* (~2°C difference) in year 5 than in year 1 and slightly cooler at *discon trt* (~2°C difference) in year 5 than in year 1. *Con dwn* experienced a smaller range of seasonal temperatures during both years (4.8–12.0°C) compared to *main ch*, *discon wood*, and *discon trt* (3.1–16.4°C). Mean daily discharge at the mouth of the Methow River was higher throughout the winter in year 5 than in year 1 (Figure 2). Peak spring flows were lower during year 5 than during year 1 (year 1, 220 m^3^ s^−1^; year 5, 190 m^3^ s^−1^).

### Community production dynamics

#### Benthic invertebrates

**FIGURE 3:**
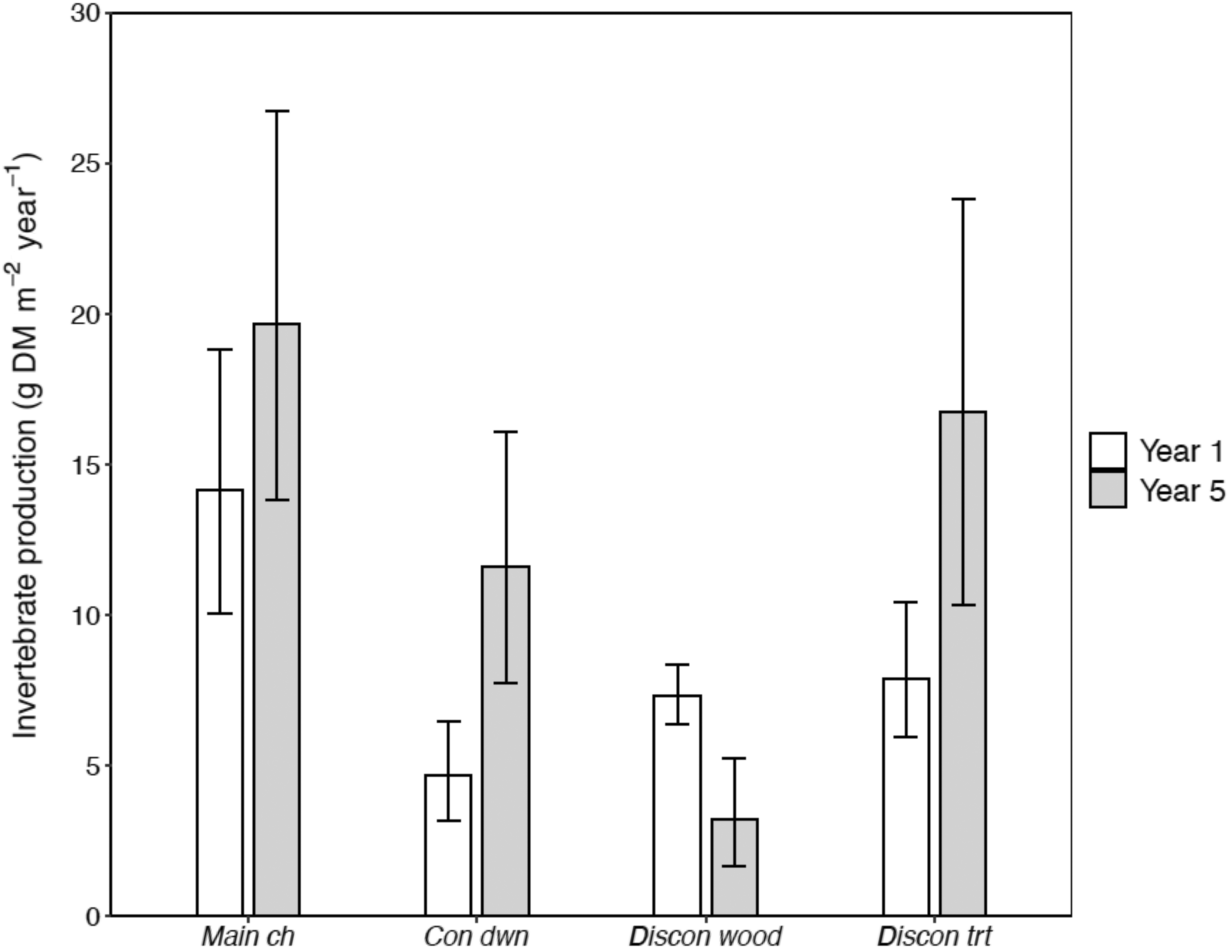
Total annual production of benthic invertebrates during study year 1 (2009–2010, white bars) and study year 5 (2015–2016, gray bars) at the main channel and side‐channel habitats of the Methow River, WA, USA. Error bars represent 95% CIs.

Total production by benthic invertebrate communities showed a greater magnitude of change between study years in side‐channel habitats than in the main channel (Figure 3). At *main ch*, annual production of invertebrates during year 1 was comparable with that of during year 5 (year 1, 14.1 g DM m^−2^ year^−1^ [95% CI: 18.9–10.0]; year 5, 19.7 g DM m^−2^ year^−1^ [95% CI: 16.1–7.7]), as 95% CIs overlapped. In contrast, invertebrate production in both reference and treated side channels either increased or decreased. Furthermore, the magnitude of change in invertebrate production observed at *discon trt* was similar to reference habitats, as invertebrate production increased ~2‐fold at both *con dwn* (year 1, 4.7 g DM m^−2^ year^−1^ [95% CI: 6.5–3.2]; year 5: 11.6 g DM m^−2^ year^−1^ [95% CI: 26.8–13.8]) and *discon trt* (year 1, 7.9 g DM m^−2^ year^−1^ [95% CI: 8.3–6.3]; year 5: 16.7 g DM m^−2^ year^−1^ [95% CI: 26.8–13.8]). At *discon wood*, the other reference side channel, invertebrate production declined 2‐fold between years (year 1, 7.3 g DM m^−2^ year^−1^ [95% CI: 6.5–3.2]; year 5: 3.2 g DM m^−2^ year^−1^ [95% CI: 5.4–1.7]).

**FIGURE 4:**
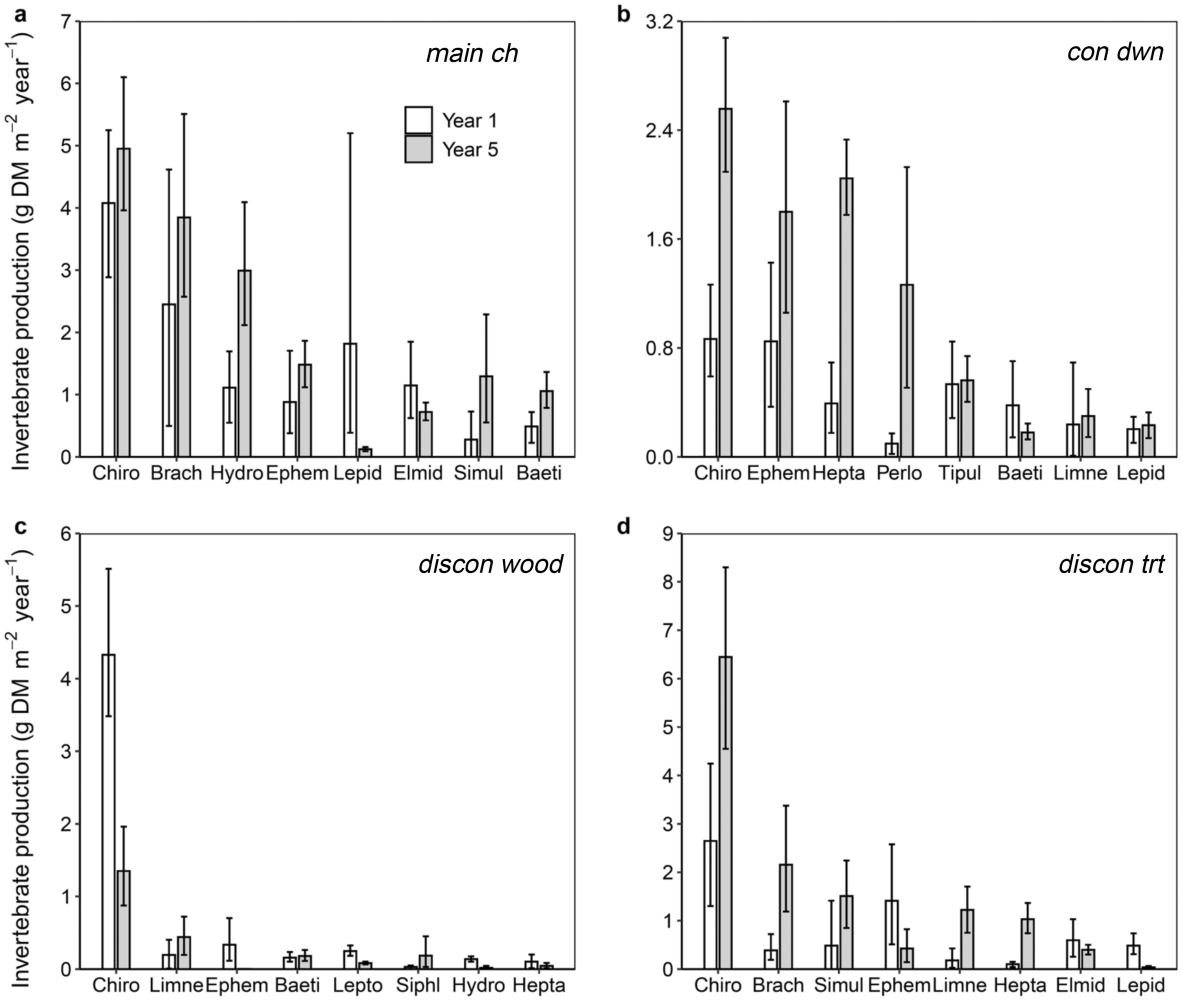
Family‐specific production of benthic invertebrates in (a) *main ch*, (b) *con dwn*, (c) *discon wood*, and (d) *discon trt* of the Methow River during study year 1 (2009–2010, white bars) and year 5 (2015–2016, gray bars). Error bars represent 95% CIs. Baeti, Baetidae (Order Ephemeroptera); Brach, Brachycentridae (Order Trichoptera); Chiro, Chironomidae (Order Diptera); Elmid, Elmidae (Order Coleopters); Ephem, Ephemerellidae (Order Ephemeroptera); Hepta, Heptageniidae (Order Ephemeroptera); Hydro, Hydropsychidae (Order Trichoptera); Lepid, Lepidostomatidae (Order Trichoptera); Lepto, Leptophlebiidae (Order Ephemeroptera); Limn, Limnephhilidae (Order Trichoptera); Perlo, Perlodidae (Order Plecoptera); Simul, Simuliidae (Order Diptera); Siphl, Siphlonuridae (Order Ephemeroptera); Tipul, Tipulidae (Order Diptera).

Changes in a few dominant invertebrate taxa largely controlled community‐wide production dynamics (Figure 4). For example, during both years, Chironomidae midges formed between 20% and 60% of total invertebrate production across habitats. At *main ch*, Chironomidae production remained similar between years (Figure 4a). At *con dwn*, however, Chironomidae production increased by 3× during year 5 (Figure 4b). Production by Heptageniidae mayflies and Perlodidae stoneflies, which formed 10% of total production, also changed dramatically between years at *con dwn*, increasing by 11× and 13×, respectively. Production by Chironomidae midges declined by 3× between years at *discon wood*, which accounted for 70% of the decrease in total invertebrate production (Figure 4c). Compared to reference sites, the magnitude of increase in Chironomidae production at the treated site was similar. At *discon trt*, Chironomidae production increased by 2.5× between study periods and was more than 3× the production of other invertebrate families during year 5 (Figure 4d). Differences in taxon‐specific production between years was largely due to differences in annual biomass (Benthic_invertebrate_biomass&production_Methow_River.xlsx in Paris, 2024).

#### Fish communities

**FIGURE 5:**
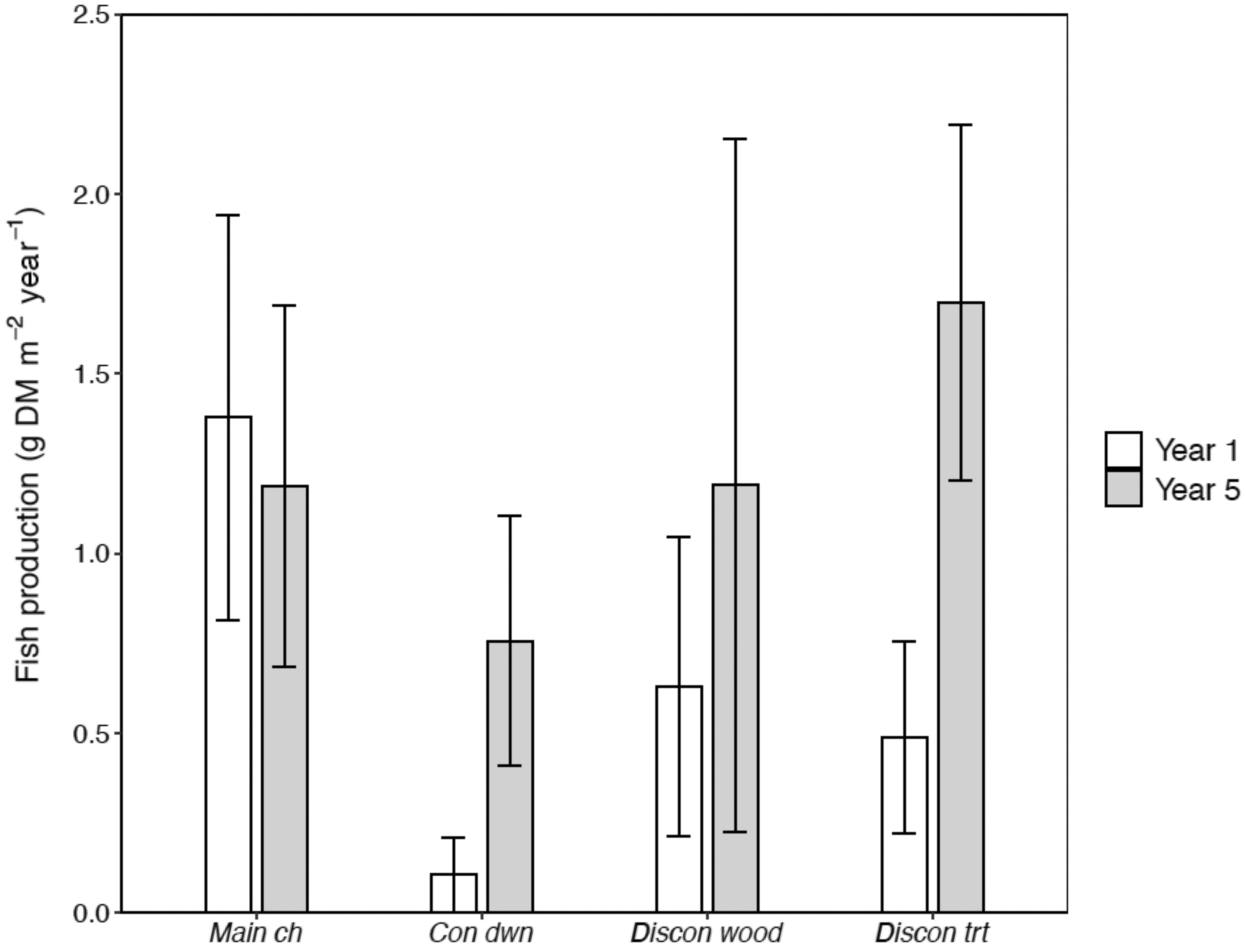
Total annual production of fish communities during study year 1 (2009–2010, white bars) and study year 5 (2015–2016, gray bars) at the main channel and side‐channel habitats of the Methow River, WA, USA. Error bars represent 95% CIs.

Mirroring interannual differences in invertebrate production, total production of fish communities expressed a greater magnitude of change in side‐channel habitats than in the main channel (Figure 5). Moreover, fish production in a reference side channel (*con dwn*) increased more dramatically than in the treated side‐channel (*discon trt*). For instance, per‐area secondary production of fish increased 7‐fold at *con dwn* (year 1, 0.1 g DM m^−2^ year^−1^ [95% CI: 0.2–0]; year 5: 0.8 g DM m^−2^ year^−1^ [95% CI: 1.1–0.4]) and 3.5‐fold at *discon trt* between years (year 1, 0.5 g DM m^−2^ year^−1^ [95% CI: 0.8–0.2]; year 5: 1.7 g DM m^−2^ year^−1^ [95% CI: 2.2–1.2]). In contrast, total fish production remained similar between years at *main ch* (year 1, 1.4 g DM m^−2^ year^−1^ [95% CI: 1.9–0.8]; year 5: 1.2 g DM m^−2^ year^−1^ [95% CI: 1.7–0.7]) and *discon wood* (year 1, 0.6 g DM m^−2^ year^−1^ [95% CI: 1.0–0.2]; year 5: 1.2 g DM m^−2^ year^−1^ [95% CI: 2.2–0.2]).

**FIGURE 6:**
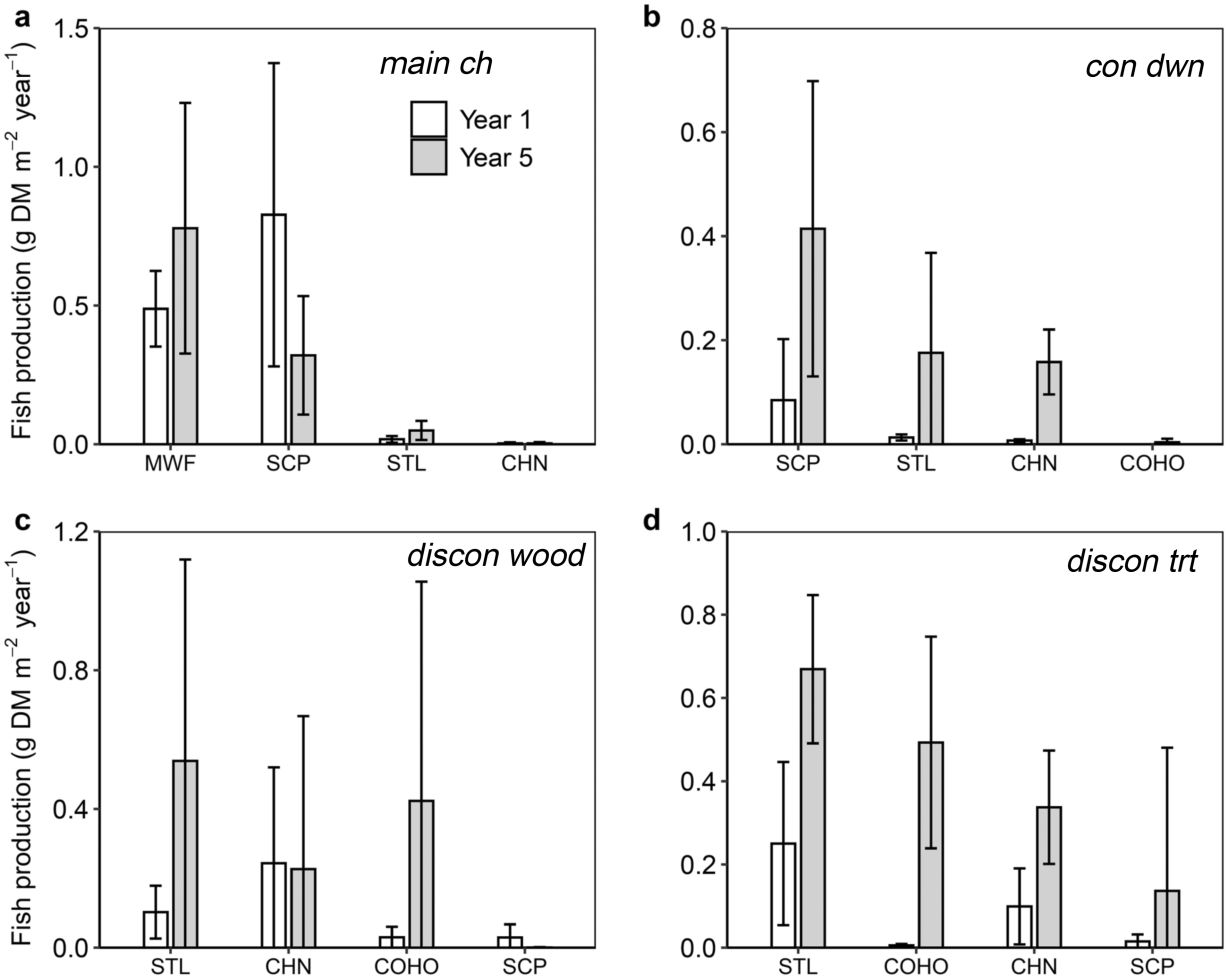
Species‐specific production of fishes in (a) *main ch*, (b) *con dwn*, (c) *discon wood*, and (d) *discon trt* of the Methow River during study year 1 (2009–2010, white bars) and study year 5 (2015–2016, gray bars). Error bars represent 95% CIs. CHN, Chinook; COHO, coho; MWF, mountain whitefish; SCP, sculpin spp.; STL, steelhead.

The production of dominant fish species in each habitat varied between years and drove community‐wide production dynamics (Figure 6). For instance, in the main channel, mountain whitefish and sculpin species formed 95% of total production during year 1 (Figure 6a). Whereas sculpin production declined by 2.6 times in year 5, whitefish production increased by 1.6 times. In the side‐channel habitats, production of sculpin species and juvenile salmonids increased during year 5 (Figure 6b–d). Sculpin formed 80% of total fish production at *con dwn* during year 1 and increased in production by 5× during year 5 (Figure 6b). At *discon wood* and *discon trt*, sculpin formed less than 5% of total fish production during year 1 but increased in absolute production by 9‐fold at *discon trt* (Figure 6c,d).

Total production of juvenile coho salmon, Chinook salmon, and steelhead trout increased in all side channel habitats, and the magnitude of change in their production was similar across reference and treated side channels (Figure 6). For example, salmonid production at *con dwn* increased from 20% to 45% of total production between years, and absolute salmonid production increased 17‐fold (Figure 6b). At *discon wood*, juvenile salmonids composed 60% of total fish production during year 1, and their absolute production increased by 3‐fold (Figure 6c). As a result of this increase, and reductions in the production of other fishes, 99% of total production at *discon wood* during year 5 was attributed to juvenile salmonids. At the manipulated *discon trt*, contributions of salmonid production to total fish production increased between years from 73% to 88%, and their absolute production increased by 4‐fold (Figure 6d). Bridgelip suckers, which were productive members of fish communities in *discon wood* and *discon trt* during year 1, were not detected in these habitats during year 5. Production dynamics were generally driven by interannual differences in fish densities across habitats (Fish_biomass&production_Methow_River.xlsx in Paris, 2024).

### Food‐web dynamics

#### Food‐web structure

**FIGURE 7:**
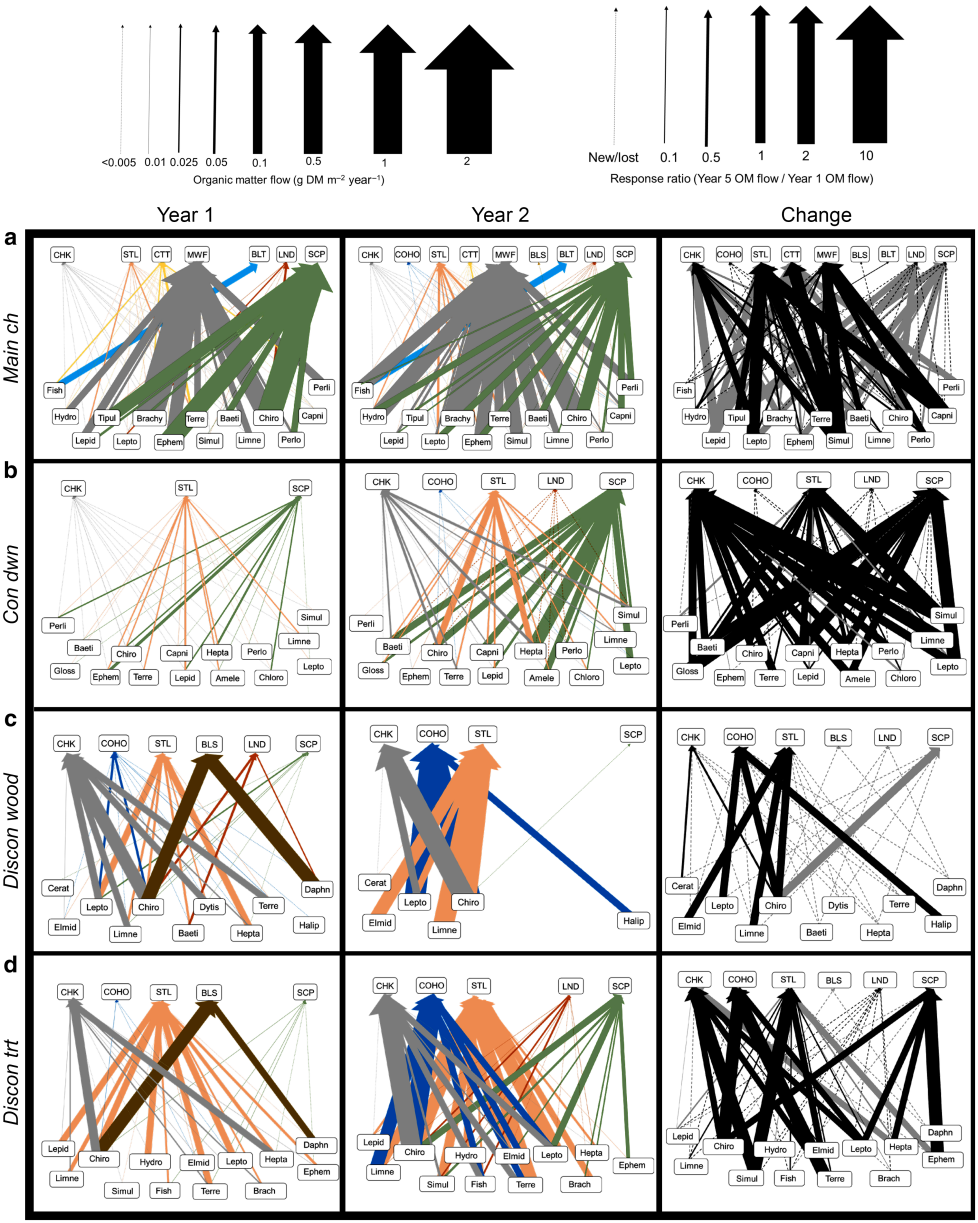
Organic matter (OM) flow food webs for the main channel side‐channel habitats of the Methow River, WA, USA, during year 1 (2009–2010) and year 5 (2015–2016) at *main ch*, *con dwn*, *discon wood*, and *discon trt*. Arrow widths represent the magnitude of organic matter flows from prey to fish consumers (see key for approximation) for year 1 and year 5 webs and the response ratios of year 5 flows to year 1 flows for the change webs. Arrow colors correspond to fish species for year 1 and year 5 webs and to relative increase (black) or relative decrease (gray) for change webs. BLS, bridgelip sucker; BLT, bull trout; CHK, Chinook salmon; COHO, coho salmon; CTT, cutthroat trout; LND, longnose dace; MWF, mountain whitefish; SCP, sculpin; STL, steelhead trout. Prey taxa are as follows: Amele, Ameletidae (Order Ephemeroptera); Baeti, Baetidae (Order Ephemeroptera); Brachy, Brachycentridae (Order Trichoptera); Capni, Capniidae (Order Plecoptera); Cerat, Ceratopogonidae (Order Diptera); Chiro, Chironomidae (Order Diptera); Chloro, Chloroperlidae; Elmid, Elmidae (Order Coleoptera); Ephem, Ephemerellidae (Order Ephemeroptera); Gloss, Glossosomatidae (Order Trichoptera); Hepta, Heptageniidae (Order Ephemeroptera); Hydro, Hydropsychidae (Order Trichoptera); Limn, Limnephhilidae (Order Trichoptera); Lepid, Lepidostomatidae (Order Trichoptera); Lepto, Leptophlebiidae (Order Ephemeroptera); Perli, Perlidae (Order Plecoptera); Perlo, Perlodidae (Order Plecoptera); Simul, Simuliidae (Order Diptera); Siphl, Siphlonuridae (Order Ephemeroptera); Terre, terrestrial arthropods; Tipul, Tipulidae (Order Diptera).

Food‐web complexity (average number of food‐web links per species) varied between the main channel and side channels during both years but changed little between years at all sites, aside from the log‐blocked side channel (*discon wood*; Figure 7). The main channel web contained more total links (~365 links vs. ~190 links), invertebrate and fish taxa (~55 taxa vs. ~44 taxa), and complexity (~6.6 average links per taxon vs. ~4.0 average links per taxon) than side‐channel food webs. Food‐web complexity in *main ch*, *con dwn*, and *discon trt* changed by less than 8% between study periods (*main ch*: year 1, 6.8, year 5, 6.4; *con dwn*: year 1, 4.0, year 5, 4.1; *discon trt*: year 1: 4.2, year 5: 4.5). In contrast, food‐web complexity at *discon wood* decreased by 2 times between study periods, driven by a 7‐fold decline in the number of food‐web links and a 3.5‐fold reduction in the number of taxa.

#### Organic matter flow

The direction and magnitude of major organic matter flows to fishes were highly variable between study years (Figure 7). Total annual organic matter flow to fishes (i.e., consumption by the fish community) was similar between study years at *main ch* (year 1, 9.8 g DM m^−2^ year^−1^ [95% CI: 13.3–5.7]; year 5, 10.4 g DM m^−2^ year^−1^ [95% CI: 15.1–5.6]; Figure 7a). However, individual flows to mountain whitefish and sculpin, which formed between 70% and 80% of total organic flow during both years, shifted from several dominant flows in year 1 to a more even distribution of flows across more prey taxa during year 5. For example, during year 1, 66% of total organic matter consumed by mountain whitefish and sculpin species came from three insect families, Chironomidae midges, Ephemerellidae mayflies, and Brachycentridae caddisflies. In contrast, flows from these three families accounted for 46% of total organic matter flow during year 5.

Organic matter flow through food webs exhibited greater dynamism in side‐channel habitats than in the main channel (Figure 7b–d). At *con dwn*, organic matter flow through the entire food web increased between year 1 and year 5 by 6‐fold (year 1, 0.6 g DM m^−2^ year^−1^ [95% CI: 0.9–0.3]; year 5, 3.9 g DM m^−2^ year^−1^ [95% CI: 5.6–2.1]; Figure 7b). Roughly 90% of the individual organic matter flows at *con dwn* present during both study years increased in absolute magnitude during year 5, and the flows that increased between time periods did so by an average of ~200×. Organic matter flow to juvenile salmonids, which formed 47%–60% of total organic matter flow during both study periods, increased by 5×. Likewise, total organic matter flow to sculpin species at this site increased 6‐fold. Total organic matter flow from Chironomidae midges, Heptageniidae mayflies, and Baetidae mayflies increased by 4.5‐fold, 14‐fold, and 11‐fold, respectively. Flows to longnose dace and juvenile coho salmon, which were not observed during year 1, appeared during year 5 at *con dwn*.

At *discon wood*, a relatively diverse set of organic matter flow pathways shifted between study years to a few flows dominated by juvenile salmonids (Figure 7c). For example, total organic matter flow to fishes at this site remained similar between study periods (year 1, 3.3 g DM m^−2^ year^−1^ [95% CI: 5.9–0.4]; year 5, 6.3 g DM m^−2^ year^−1^ [95% CI: 12.7–0.1]), but the total number of individual flows between fishes and their prey decreased 7‐fold (188 vs. 28). Flows to bridgelip sucker, which formed 30% of total organic matter flow during year 1, disappeared during year 5. Total organic matter flow to juvenile coho increased by 14‐fold between years, and the relative contribution of juvenile salmonid consumption to total organic matter flow shifted from 62% during year 1 to 99% during year 5. However, juvenile salmon and steelhead consumed 6× fewer prey items between study periods, and during year 5, flows from Chironomidae midges, Limnephilidae caddisflies, and Leptophlebiidae mayflies formed 90% of total organic matter flow.

The food web of the treated site *discon trt* also showed dramatic variation between years in both patterns and magnitudes of organic matter flow, yet food‐web changes involving juvenile salmonids were comparable to changes observed in reference sites (Figure 7d). For instance, total organic matter flow to fishes at this site increased by 4‐fold (year 1, 2.6 g DM m^−2^ year^−1^ [95% CI: 4.5–0.8]; year 5, 9.4 g DM m^−2^ year^−1^ [95% CI: 12.7–6.2]), which was similar to *con dwn* (6‐fold increase). In addition, organic matter flow to juvenile salmon and steelhead trout at *discon trt* increased by 4‐fold between years, comparable with that at *con dwn* (5‐fold difference). Organic matter flows to bridgelip sucker (20% of total flow during year 1) were replaced by flows to coho salmon (28% of total flow during year 5), which was also observed in *discon wood*. Also, increases in large flows from Chironomidae midges (7× increase), Limnephilidae caddisflies (5× increase), and terrestrial arthropods (4× increase) to juvenile salmonid consumption at this site were all within the ranges observed at the reference side channels (between 3× and 10× increases).

#### Ecotrophic efficiency

**FIGURE 8:**
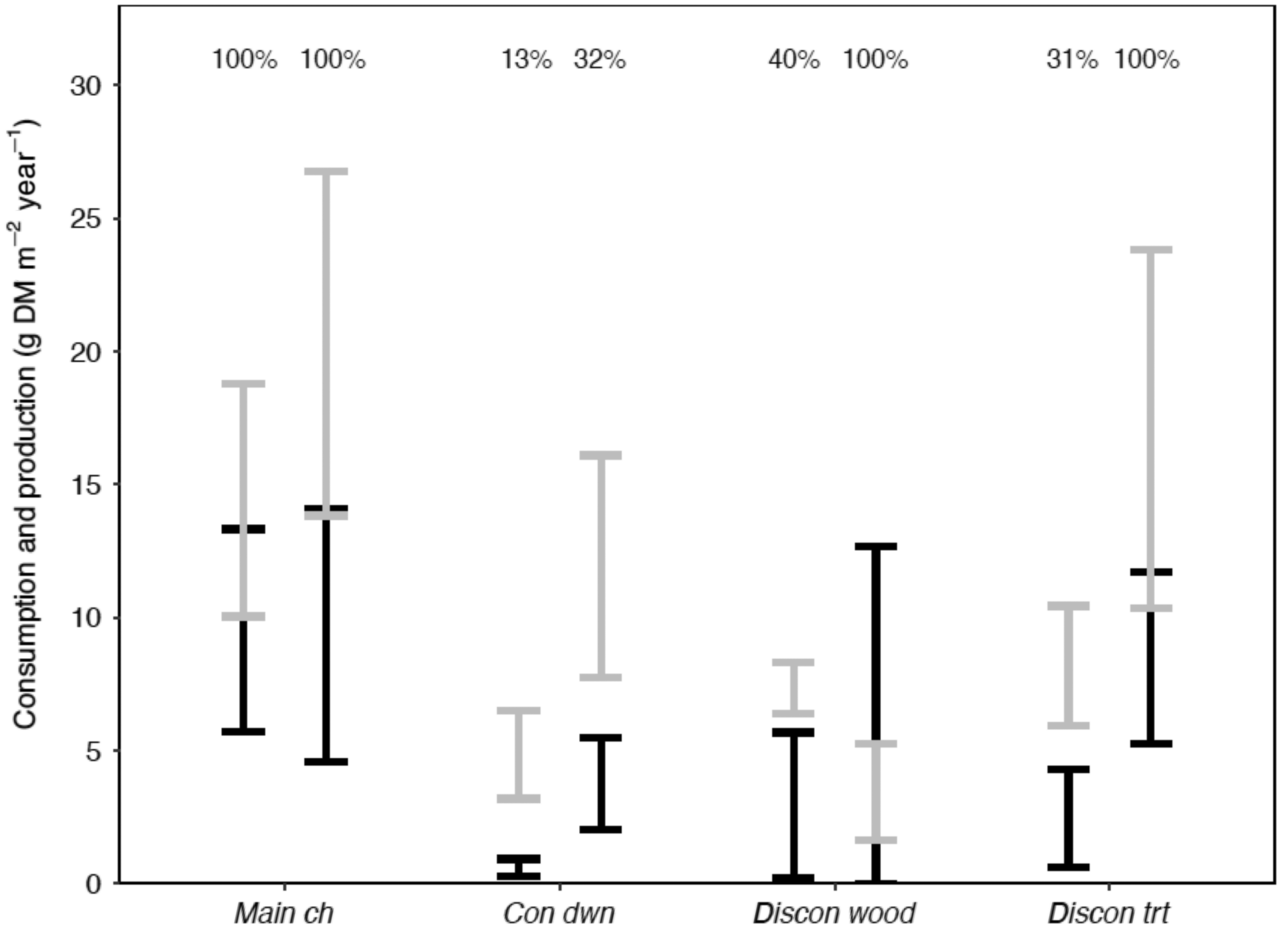
Comparisons of total benthic invertebrate consumption by fish (black bars) to total benthic invertebrate production (gray bars) during year 1 (2009–2010, left bars) and year 5 (2015–2016, right) in the main channel and side‐channel habitats of the Methow River floodplain, WA, USA. Ranges represent 95% CIs calculated via bootstrapping. Percentages at the top of the plot represent the proportion of invertebrate production consumed by fishes during each study year, with instances of overlapping CIs representing 100% of invertebrate production consumed by fishes.

Fish communities in side‐channel habitats consumed a greater fraction of benthic prey production during year 5 than during year 1 (Figure 8). At *main ch*, the consumption of benthic invertebrate production by the fish community was highly efficient during both study periods (i.e., overlapping 95% CIs of fish consumption and prey production). In contrast, ecotrophic efficiency (i.e., proportion of prey production consumed by the fish community) at both reference and manipulated side channels increased by at least 2‐fold between year 1 and year 5. At *con dwn*, ecotrophic efficiency increased from 13% to 32% from year 1 to year 5. At *discon wood*, efficiency increased from 40% in year 1 to 100% in year 5, as fish consumption completely overlapped with benthic prey production during year 5, implying little to no surplus of annual benthic production. Similarly, in *discon trt*, ecotrophic efficiency increased from 31% in year 1 to 100% during year 5.

#### Interaction strengths

**FIGURE 9:**
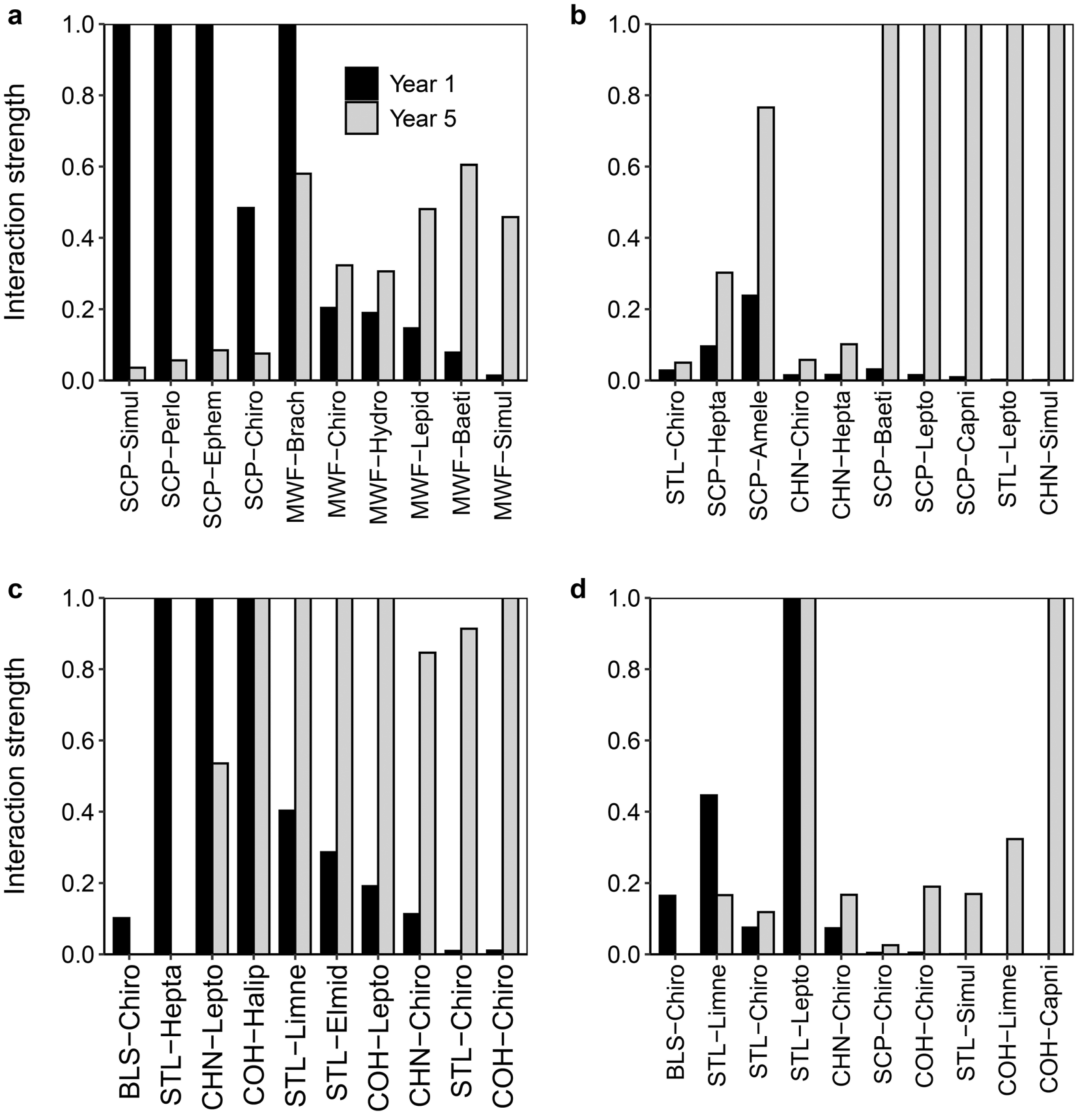
Interaction strengths of dominant organic flows between fish and benthic invertebrates in (a) *main ch*, (b) *con dwn*, (c) *discon wood*, and (d) *discon trt* of the Methow River during year 1 (2009–2010, white bars) and year 5 (2015–2016, gray bars). See legends of Figures 4 and 6 for taxa abbreviations. Interactions are arranged from left to right as those that were relatively stronger in year 1 (left) to those that were stronger in year 5 (right).

Interaction strengths involving the dominant organic matter flows between fishes and benthic prey taxa were highly variable between study periods, with weak interactions often intensifying and strong interactions often weakening (Figure 9). For example, of the 40 dominant flows across study sites, the interaction strength (i.e., proportion of taxon‐specific, annual production consumed by a fish species) of 38 flows changed by at least 2× between study periods. In fact, the interaction strengths of 35% of the dominant food‐web links across sites changed by nearly 9‐fold between study periods. Furthermore, 54% of the interaction strengths that were <0.4 during year 1 switched to >0.6 during year 5. Reciprocally, 75% of the interaction strengths that were >0.6 during year 1 shifted to <0.4 during year 5.

## DISCUSSION

Our findings are consistent with previous research demonstrating that river‐floodplains support high levels of spatial and temporal variation in food‐web structure and function (Junk et al., 1989; Stanford et al., 2005; Tockner et al., 2000; Winemiller, 1990). We show that spatial heterogeneity in food‐web complexity mediates food‐web dynamics across habitat mosaics of river‐floodplains, consistent with landscape‐level theory (see Rooney et al., 2008; Rooney & McCann, 2012), as the more speciose and complex main channel food web was more temporally invariant, whereas the less diverse and less complex side channel webs were more dynamic. Furthermore, the temporal variation in benthic prey production, fish consumption, organic matter flows, and food‐web interaction strengths within multiple side‐channel habitats surpassed food‐web responses to side‐channel habitat manipulation via engineered logjams. A potential consequence of this food‐web variability was that increased prey production within side‐channel habitats may have fueled elevated densities of juvenile salmon. Thus, food‐web dynamism across river‐floodplains, particularly in side‐channel habitats, showed the capacity to overshadow effects of habitat engineering. Rather than being considered statistical noise (i.e., obscuring the effects of restoration efforts), this variability in food‐web function may be a vital component of free‐flowing and dynamic riverscapes, buffering biodiversity and consumer production fishes during times of environmental change.

### Food‐web theory and dynamics across the floodplain mosaic

Our results were consistent with theoretical treatments of food webs that integrate landscape heterogeneity and food‐web dynamics. Rooney et al. (2006, 2008), Rooney and McCann (2012) predict that spatial differences in food‐web complexity across habitat patches create spatially variable dynamics in energy and material flow. Patches with more species and complex food webs appear more resistant to environmental change (i.e., basal food‐web productivity, loss of species), as material and energy flow is diffused among many taxa and links (Rooney et al., 2008; Rooney & McCann, 2012). In contrast, simple food webs with fewer feeding links become unstable (Rooney et al., 2006). We observed that dynamics of material flow through food webs across the Methow River floodplain mosaic were linked to main‐channel versus side‐channel differences in food‐web complexity. The food web of the main channel during both study years comprised ~2× more total links, ~30% more fish and invertebrate taxa, and ~50% more links per taxon. These spatial patterns in food‐web complexity were associated with food‐web stability as total organic matter flow through the main‐channel food web only slightly changed between study years. However, in the *con dwn* and *discon trt* side channels, ~4.5× more organic matter flowed through food webs during year 5 than year 1. Furthermore, the spatial heterogeneity in food‐web complexity and dynamics that we observed could contribute to the long‐term persistence (i.e., stability) of communities when the spatially distinct food webs are linked by foraging consumers moving among habitat patches (Rooney et al., 2006; Rooney & McCann, 2012). At present, we have little understanding of how fishes moving between the main channel and side‐channel habitats may link food webs in the Methow River. In other floodplains, fishes commonly move among and feed within a wide range of riverine and floodplain habitats, particularly during flood stage (Balcombe et al., 2005; Kwak, 1988; Winemiller & Jepsen, 1998), which suggests that future investigations should examine the degree to which mobile fish consumers link main‐channel and side‐channel food webs and how such coupling regulates riverscape food‐web dynamics.

We also observed substantial variability in food‐web interaction strengths across the Methow River floodplain mosaic. In general, not all interaction strengths increased or decreased consistently between study years. Rather, many strong interactions became weaker, many weak interactions became stronger, and the identity of each interaction (i.e., fish and invertebrate taxa involved) that shifted strength varied across the habitat mosaic. Weak interactions are predicted to confer stability and promote biodiversity, as these interactions offset the volatile dynamics found in strong feeding interactions via adaptive prey switching by consumers (Kondoh, 2003; McCann, 2000; McCann et al., 1998). Moreover, spatial heterogeneity of interaction strength and identity can reduce average interaction strengths across the floodplain mosaic when aggregated (Bellmore et al., 2015). Thus, the fluidity in food‐web interaction strengths that we observed across the habitat mosaic may also promote species coexistence in the Methow River riverscape.

We speculate that biophysical processes occurring at multiple spatial scales drove the food‐web dynamics of side‐channel communities. First, changes in the production of side‐channel fish communities during year 5 were linked to increased densities of one of the dominant fish taxa, juvenile salmonids, which were in turn correlated with elevated adult salmon returns to the Methow River watershed. Returns of Chinook and coho salmon were greater in the year preceding year 5 (2014) than the year preceding year 1 (2008), as Chinook salmon and coho salmon built 2.4× and 4.5× more redds, respectively, in the main channel during year 5 (Snow et al., 2017; G. Watson, personal communication). Second, we speculate that interannual differences in watershed discharge influenced invertebrate biomass and production. During year 5, flow through the Methow River watershed was greater in the winter and showed a lower peak flow than during year 1. Because invertebrate densities and biomass can be reduced via ice development during winter low flows (Martin et al., 2001) and during high flow events that shear standing biomass (Kendrick et al., 2019; Whiles & Wallace, 1995), yearly differences in flow timing and magnitude may have improved winter‐ and spring‐time conditions for aquatic invertebrates in fringing side‐channel habitats in year 5. Finally, natural, large‐wood dynamics may have suppressed secondary production in one side‐channel habitat. Between year 1 and year 5, a logjam ~330 m^2^ in area blocked the upstream entrance of *discon wood*. Natural wood dynamics can be an agent of local habitat change in gravel‐bed river‐floodplains (Latterell & Naiman, 2007; Naiman et al., 2010) as floods lodge large wood in floodplain channels, blocking the flow of water and gravel and affecting habitat structure and quality (Wohl, 2013)—thus influencing species interactions. Between sampling years, benthic invertebrate production at *discon wood* declined, yet fish production remained similar, leading to fish consuming all or nearly all the annual invertebrate production. Taken together, spatially nested biophysical filters—namely out‐of‐basin salmon survival, basin‐scale patterns of flow regime, and localized wood transport—may have influenced food‐web dynamics in side‐channel habitats.

### Dynamic food webs, habitat restoration, and salmon

The range of natural variation in food‐web dynamics among sites surpassed food‐web effects of the side‐channel engineering. Our results showed that food‐web productivity of floodplain habitats varied through time, as invertebrate production and production and consumption by fishes increased between study years in both a non‐manipulated side channel and treated side‐channel habitat. In fact, changes in secondary production and organic matter were generally of greater magnitude at reference *con dwn* than at the manipulated *discon trt*. Thus, we inferred that the food‐web changes at *discon trt* could not be attributed to the engineered logjams alone and that natural dynamics in species growth, abundance, and feeding interactions were also key in driving temporal patterns in secondary production. This style of food‐web study and its methods, however, possess inherent difficulties due to variability in space, time, and measurement. To examine how these uncertainties might influence interpretation of our findings, we conducted a simple thought experiment. For both sampling periods at *con dwn*, we iteratively widened the range of 95% CIs around total production estimates for invertebrates and fishes by 1% until the intervals overlapped across sampling periods, thus simulating the magnitude of error that would have been necessary to assert that no change occurred at this non‐manipulated side channel. We observed that the simulated CIs for invertebrate and fish production would need to have differed from the empirical, bootstrapped intervals by 27% and 33%, respectively, in order to alter our findings. In other words, uncertainty around production estimates would need to be approximately one‐third greater. This simulation suggests that substantial errors in our estimates would have been necessary to override the study's ability to detect change, which we judge strengthens the inference that natural food‐web dynamics were of greater magnitude than the effects of engineered logjams.

Although other studies have suggested a variety of complexities in quantitatively evaluating fish population response to local restoration (i.e., fish movement between habitats: Gowan & Fausch, 1996; Polivka & Claeson, 2020; scale of restoration: Roni et al., 2010), our findings suggest that a potentially underappreciated factor may be the dynamic food webs within which fishes participate. Food‐web dynamism could be interpreted as “noise” that obscured responses to habitat manipulation in the Methow River. An alternative interpretation, however, is that this “shifting food web mosaic” is an intrinsic component of river‐floodplains in free‐flowing watersheds, essential to creating and maintaining productive and stable fish populations. This would be especially true if prey productivity and food‐web energy flows vary out‐of‐phase from one another in different habitat patches (Bellmore et al., 2022; Rossi et al., 2024), whereby decreases in productivity in some patches are offset by increases in other patches creating the so‐called portfolio effects (Moore et al., 2014; Schindler et al., 2010). For example, in year 5 (2015–2016), invertebrate production was higher in two out of three side channel habitats. Greater prey availability in turn supported higher fish consumption necessary to sustain larger populations of juvenile salmonids. Food‐web variability was most striking in side‐channel habitats, suggesting that these environments may play a disproportionate role in absorbing interannual variation in populations and sustaining salmon and steelhead in the long term. It should be noted that salmon and steelhead abundances in the Methow Basin, and throughout their range, were historically much higher and interannual swings likely more extreme (see Bisson et al., 2009). This suggests food‐web dynamism in side‐channel habitats should be evaluated in free‐flowing river systems with near‐historic runs of salmon and steelhead trout to explore if dynamics in community and food‐web interactions within off‐channel habitats can buffer salmon populations.

Our findings emphasize the need to evaluate how habitat enhancement projects are assessed in free‐flowing riverscapes, especially those that maintain dynamic habitats and associated food webs. In these contexts, it may be impractical to assess population responses to individual projects, and more focus should be placed on detecting ecologically relevant signals at broader spatial scales that integrate responses to numerous habitat restoration projects (Bennett et al., 2016; Zimmerman et al., 2012). For endangered salmon, this could include examining long‐term trends in the number, size, and condition of salmon smolts produced by a given number of spawners at the watershed scale. Furthermore, it is likely that variation in salmon populations is—in part—derived from spatiotemporal dynamism in the food‐web pathways that support salmon at both local and watershed scales (Jeffres et al., 2020; Pess et al., 2012; Phillis et al., 2018). Our results emphasize that understanding of the range of community and food‐web dynamics naturally occurring in salmon‐bearing watersheds of the Pacific Northwest should be illuminated before habitat manipulation projects can be properly assessed. Understanding current and predicted impacts to food‐web structure and dynamics is especially important as floodplain restoration projects increase in spatial scale from restoring specific features to “resetting” entire floodplain valley bottoms (i.e., “stage 0 restoration”; Flitcroft et al., 2022; Jennings et al., 2023).

Watershed‐scale processes that create and maintain spatially heterogenous and dynamic habitat are receiving greater attention in river restoration (Beechie et al., 2010; Kondolf et al., 2006; Palmer et al., 2005; Wohl et al., 2015). General concepts surrounding process‐based river restoration often focus on the creation and maintenance of physical habitat, even though food‐web interactions directly and indirectly influence the outcomes of river restoration with important repercussions for species of interest (Bellmore et al., 2017; Whitney et al., 2020). Therefore, efforts to restore and maintain biodiversity in riverscapes, especially with regard to variable salmon and steelhead numbers in the Pacific northwest, should expand the conceptual framework of process‐based restoration to embrace the dynamic characteristics of food webs.

## CONFLICT OF INTEREST STATEMENT

The authors declare no conflicts of interest.

## Supporting information


Appendix S1.

Appendix S2.

## Data Availability

Data (Paris, 2024) are available in Figshare at https://doi.org/10.6084/m9.figshare.25238527.v1.
