# Supplementary material for: Food‐web dynamics of a floodplain mosaic overshadow the effects of engineered logjams for Pacific salmon and steelhead
Source: Ecol Appl. 2024 Dec 3;35(1):e3076. doi: 10.1002/eap.3076 (PMC11731428; doi:10.1002/eap.3076)

James C. Paris, Colden V. Baxter, J. Ryan Bellmore, Joseph R. Benjamin. Food-web dynamics of a floodplain mosaic overshadow the effects of engineered logjams for Pacific salmon and steelhead. Ecological Applications.

Appendix S1

Figure S1. Photographs of side-channel habitats included in comparative food-web analysis during study year 1 (2009-2010) and study year 5 (2015-2016) in the Methow River, WA. Credit: J. Ryan Bellmore, James C. Paris

Con dwn, Year 1 (2009-2010)

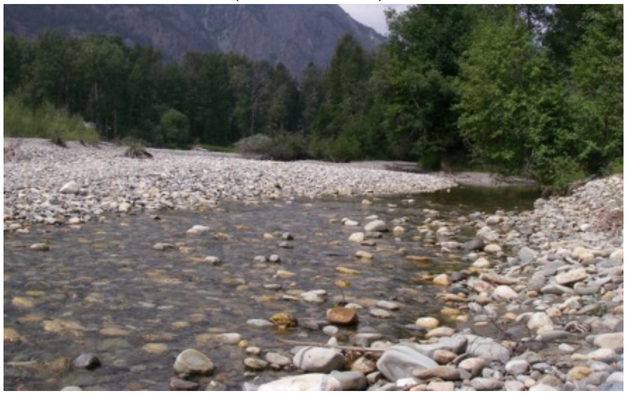

Con dwn, year 5 (2015-2016)

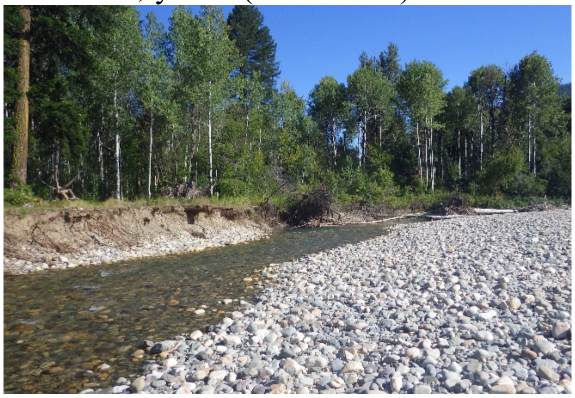

Discon wood, year 1 (2009-2010)

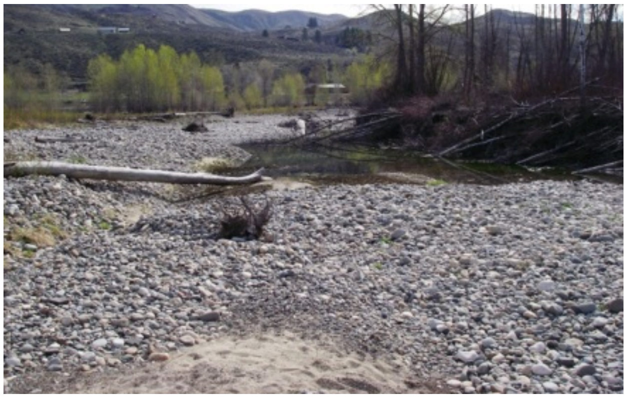

Discon wood, year 5 (2015-2016)

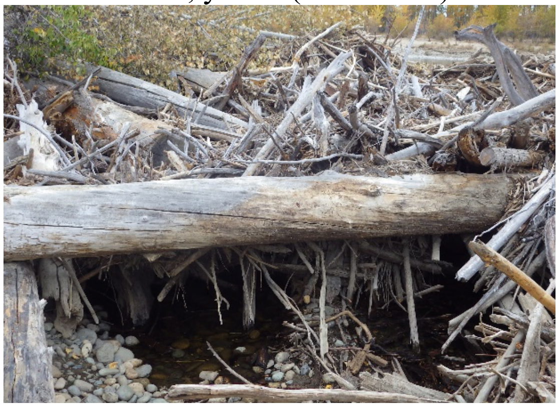

Discon trt, year 1 (2009-2010)

Discon trt, year 5 (2015-2016)

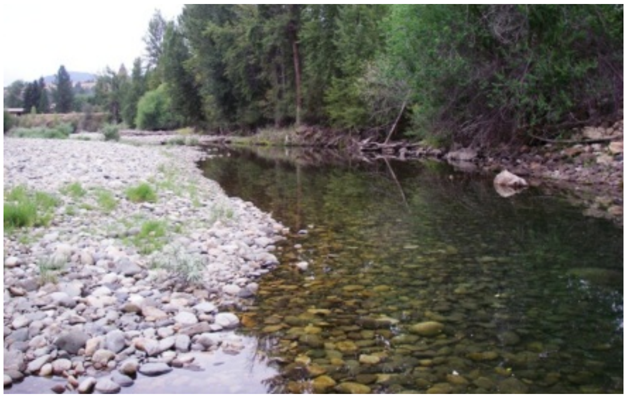

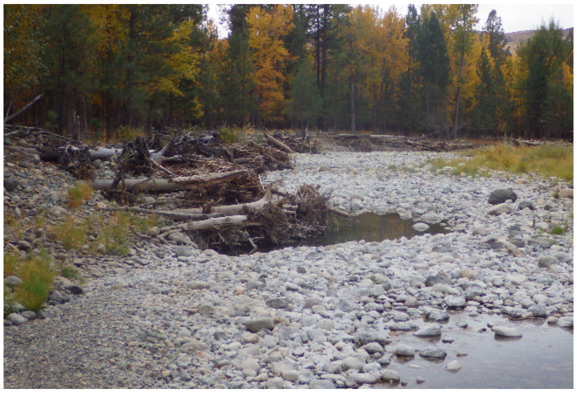

Supplement: Supplementary file 1 — Appendix S1. [file EAP-35-e3076-s002.pdf]
