# Supplementary material for: Food‐web dynamics of a floodplain mosaic overshadow the effects of engineered logjams for Pacific salmon and steelhead
Source: Ecol Appl. 2024 Dec 3;35(1):e3076. doi: 10.1002/eap.3076 (PMC11731428; doi:10.1002/eap.3076)
Supplement: Supplementary file 2 — Appendix S2. [file EAP-35-e3076-s001.pdf]

James C. Paris, Colden V. Baxter, J. Ryan Bellmore, Joseph R. Benjamin. Food-web dynamics of a floodplain mosaic overshadow the effects of engineered logjams for Pacific salmon and steelhead. Ecological Applications.

Appendix S2

Table S1. Average seasonal water temperature (°C) of the main channel and side-channel habitats of the Methow River, WA, during study year 1 (2009-2010) and study year 5 (2015-2016).

| | | Average temp (°C) | |
|---|---|---|---|
| Site | Season | Year 1 | Year 5 |
| *Main ch* | Summer | 15.2 | 16.4 |
| | Fall | 4.6 | 6.7 |
| | Winter | 3.1 | 4.8 |
| *Con dwn* | Summer | 11.4 | 12 |
| | Fall | 6.7 | 8.7 |
| | Winter | 5.1 | 4.8 |
| *Discon wood* | Summer | 16.1 | 15.7 |
| | Fall | 4.7 | - |
| | Winter | - | - |
| *Discon trt* | Summer | 14.9 | 15.9 |
| | Fall | 7.4 | 11 |
| | Winter | 5.4 | 3.1 |
